# Correction: Deng et al. Assessment of the Macrophage Scavenger Receptor CD163 in Mediating *Glaesserella parasuis* Infection of Host Cells. *Vet. Sci.* 2023, *10*, 235

**DOI:** 10.3390/vetsci10070458

**Published:** 2023-07-12

**Authors:** Xiangwei Deng, Shuilian Li, Ying Zhu, Bo Yu, Jing Zhang, Qianhai Fang, Zhimin Li, Hongbo Chen, Huanhuan Zhou

**Affiliations:** Laboratory of Genetic Breeding, Reproduction and Precision Livestock Farming & Hubei Provincial Center of Technology Innovation for Domestic Animal Breeding, School of Animal Science and Nutritional Engineering, Wuhan Polytechnic University, Wuhan 430023, China

## Error in Figure

In the original publication [1], there was a mistake in **Figure 2** as published. **Specifically, in the two pictures in Figure 2C, showing the results of the adhesion study using a Scanning Electron Microscope (SEM), the same original SEM picture was inadvertently used.** The corrected **Figure 2** appears below. The authors state that the scientific conclusions are unaffected. This correction was approved by the Academic Editor. The original publication has also been updated.

## Figures and Tables

**Figure 2:**
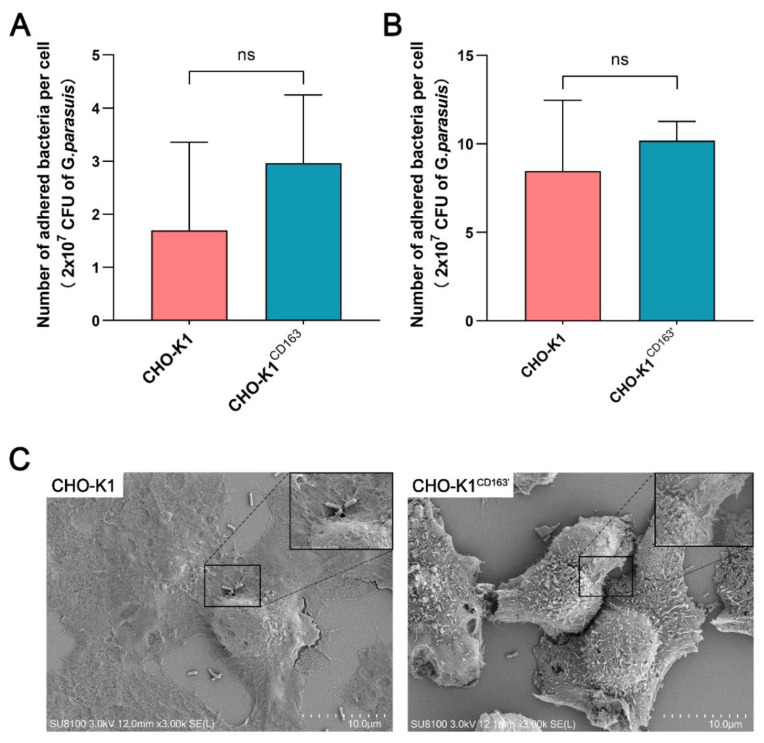
The adhesion of *G. parasuis* to CHO-K1 cells with or without CD163 expression. (**A**,**B**) The average number of adhered *G. parasuis* per cell at 6 h incubation. The bacterial inoculum tested was 2 × 10^7^ CFU. (**C**) SEM micrograph showing the attachment of *G. parasuis* to the surface of CHO-K1 cells or CHO-K1^CD163′^ cells after incubation for 6 h. CHO-K1^CD163^: porcine CD163 overexpressed in CHO-K1 cells by transient transfection. CHO-K1^CD163′^: CHO-K1 cells stably overexpressing porcine CD163. ns: Nonsignificant.
