# Supplementary material for: Assessment of the Macrophage Scavenger Receptor CD163 in Mediating Glaesserella parasuis Infection of Host Cells
Source: Vet Sci. 2023 Mar 21;10(3):235. doi: 10.3390/vetsci10030235 (PMC10054613; doi:10.3390/vetsci10030235)
Supplement: Supplementary file 1 [file vetsci-10-00235-s001.zip › vetsci-2261517-supplementary.pdf]

Supplementary Table S1. Names of genes and primers used for Q-PCR analysis.

Gene	Accession number	Primers (5′-3′)	Product size (bp)	Annealing Temperature (℃)
RPL32	NM_001001636.1	F: CGGAAGTTTCTGGTACACAATGTAA<br>R: TGGAAGAGACGTTGTGAGCAA	94	58–60
β-actin	XM_027429179.2	F: CGGAAGTTTCTGGTACACAATGTAA<br>R: TGGAAGAGACGTTGTGAGCAA	94	59
IL-4	XM_003498480.2	F: CCACGGAGAAAGACCTCATCTG<br>R: GGGTCACCTCATGTTGGAAATAAA	72	60
IL-6	XM_027429568.2	F: AGTCGGAGGTTTGGTTACACA<br>R: TTAGGGTTTTGGTGGTGCTCT	299	59
IL-10	XM_027417709.2	F: CGAGAGCTGAGAACTGCCTT<br>R: CCTGGGGCATCACTTCTACC	169	60
TGF-β	XM_027402047.2	F: GACCTGCCCTGGAAAAGAAGT<br>R: ACTCATCTCTTCTGGCCAAGC	148	60
INF-γ	XM_035439009.1	F: ACACGTTGCTTCTTGGCTTT<br>R: TCCATTCCCAACATCTTCT	264	58
